# Spinal Muscle Health in Chronic Low Back Pain: An Integrated Narrative Review of Muscle Quality, Function, and Clinical Implications

**DOI:** 10.3390/bioengineering13091068

**Published:** 2026-09-14

**Authors:** Massimo Rossi, Gabriele Capo, Ali Baram, Mario De Robertis, Leonardo Anselmi, Donato Creatura, Generoso Farinaro, Federico Pessina, Maurizio Fornari, Carlo Brembilla

**Affiliations:** 1 Scuolaecomskbo, Istituto Ortopedico Rizzoli, Via Giulio Cesare Pupilli 1, 40100 Bologna, Italy; 2 Department of Neurosurgery, IRCCS Humanitas Research Hospital, Via Alessandro Manzoni 56, 20089 Rozzano, Italy; 3 Department of Biomedical Sciences, Humanitas University, Via Rita Levi Montalcini 4, 20090 Pieve Emanuele, Italy

**Keywords:** chronic low back pain, spinal muscle health, paraspinal muscles, muscle quality, fatty infiltration, neuromuscular function, rehabilitation, patient phenotyping

## Abstract

Chronic low back pain (CLBP) is a leading cause of disability and is characterized by marked clinical heterogeneity. Structural imaging remains fundamental for diagnosis and treatment planning, yet anatomical abnormalities alone do not fully explain differences in pain, disability, physical performance, or treatment response. Increasing evidence implicates paraspinal muscle morphology, composition, neuromuscular behavior, endurance, and physical conditioning as complementary determinants of spinal function. This narrative review synthesizes current evidence within the concept of spinal muscle health, defined here as a multidimensional, region-specific construct reflecting the structural, compositional, neuromuscular, and functional capacity of the muscles contributing to spinal control and load management. The construct is not proposed as a new diagnosis or as a substitute for established concepts such as sarcopenia, myosteatosis, or deconditioning; rather, it provides an integrative framework for considering complementary muscle-related domains that cannot be represented adequately by a single imaging or performance measure. We review muscle-specific and level-specific findings, measurement approaches, sources of heterogeneity, longitudinal and interventional evidence, and the interaction of muscle-related features with systemic and psychosocial factors. Current evidence supports associations between selected paraspinal muscle abnormalities and CLBP, particularly for multifidus composition, but findings vary substantially across muscles, spinal levels, populations, and measurement methods. Most evidence remains cross-sectional, and causal direction is uncertain. Clinical improvement after exercise may occur without consistent normalization of imaging-derived muscle composition. Accordingly, spinal muscle health is best regarded at present as a research and clinical reasoning framework rather than a validated diagnostic phenotype. Standardized measurement, longitudinal studies, and intervention trials linking changes in muscle-related parameters to patient-centered outcomes are required before routine clinical implementation.

## 1. Introduction

Chronic low back pain (CLBP) remains one of the leading causes of disability worldwide and represents a major public health challenge, with substantial socioeconomic consequences and increasing prevalence across age groups [1]. Despite advances in imaging, surgical techniques, and rehabilitation, CLBP remains remarkably heterogeneous. Patients with apparently similar structural abnormalities may experience profoundly different levels of pain, disability, physical performance, and response to treatment.

Conventional imaging is indispensable for identifying degenerative changes, neural compression, instability, deformity, and other structural abnormalities that guide clinical decision making. However, imaging findings show only modest associations with symptom severity, and degenerative changes are common in asymptomatic individuals, particularly with advancing age [2,3]. CLBP is therefore increasingly understood within a multidimensional biopsychosocial framework in which structural, biological, functional, behavioral, and psychosocial factors interact [4].

Within this framework, attention has increasingly focused on the active spinal stabilizing system. Systematic reviews have associated CLBP with alterations in paraspinal muscle size, composition, fatty infiltration, neuromuscular control, and physical performance [5,6]. These dimensions have often been investigated separately, however, and their biological and clinical meaning is complicated by differences in muscle anatomy, spinal level, age, sex, body composition, physical activity, underlying spinal pathology, and measurement methodology.

This narrative review integrates evidence on paraspinal muscle morphology, muscle quality, neuromuscular function, endurance, and functional reserve within the broader concept of spinal muscle health. We aim to (1) define the construct operationally and distinguish it from related concepts; (2) critically appraise the consistency and limitations of the evidence; (3) summarize feasible measurement approaches and their current limitations; and (4) examine potential implications for patient phenotyping and rehabilitation while explicitly separating current evidence from hypotheses requiring prospective validation.

## 2. Methods: Narrative Literature Search

This article was designed as an integrated narrative review rather than a systematic review or meta-analysis. To ensure an up-to-date evidence base, targeted searches were performed in PubMed/MEDLINE through August 2026, supplemented by reference-list screening of relevant systematic reviews and key primary studies. Search concepts combined terms related to low back pain (“low back pain”, “chronic low back pain”) with terms related to paraspinal musculature and assessment (“paraspinal muscle”, “multifidus”, “erector spinae”, “psoas”, “quadratus lumborum”, “muscle quality”, “fatty infiltration”, “myosteatosis”, “cross-sectional area”, “MRI”, “CT”, “ultrasound”, “EMG”, “motor control”, “sarcopenia”, “exercise”, and “rehabilitation”). English-language human studies were considered, with emphasis on systematic reviews and meta-analyses, consensus statements, longitudinal studies, interventional trials, and recent methodological studies. Seminal biomechanical and motor-control publications were retained where necessary to establish the conceptual background. Study selection was purposive and relevance-based, consistent with a narrative synthesis; no formal risk-of-bias assessment or quantitative pooling was performed. Accordingly, the review should not be interpreted as an exhaustive systematic evidence synthesis.

## 3. The Active Spine: A Functional Concept

Traditional approaches to CLBP have primarily emphasized structural pathology involving discs, facet joints, ligaments, alignment, and neural elements. Contemporary concepts additionally recognize that spinal stability and function emerge from continuous interaction between passive anatomical structures and active neuromuscular systems.

Panjabi’s model of spinal stability describes an integrated system consisting of passive structures, active muscular components, and neural control mechanisms [7]. Within this model, spinal muscles contribute to stability, movement control, and adaptation to changing mechanical demands. McGill subsequently emphasized the contribution of muscular function to spinal stiffness, load sharing, and mechanical resilience [8].

Motor control research further demonstrated altered anticipatory trunk muscle activation in individuals with low back pain [9,10]. Such findings shifted attention from muscle size alone toward timing, recruitment, coordination, and task-specific behavior. The active spine concept therefore provides a useful foundation for interpreting contemporary evidence on muscle quantity, tissue composition, neuromuscular competence, endurance, and functional reserve, while remaining compatible with broader biopsychosocial models of CLBP.

## 4. Defining Spinal Muscle Health

We define spinal muscle health as a multidimensional, region-specific construct reflecting the structural, compositional, neuromuscular, and functional capacity of the muscles contributing to spinal control and load management. It is not intended as a diagnostic entity, a new disease label, or a replacement for established constructs. Its proposed value is integrative: muscle quantity, muscle quality, neuromuscular competence, endurance, and conditioning provide complementary information and should not be assumed to be interchangeable.

This definition differs from paraspinal muscle degeneration, which generally emphasizes structural or compositional abnormalities; from myosteatosis, which specifically concerns pathological fat accumulation and impaired muscle quality; from sarcopenia, which is a systemic skeletal muscle disorder; and from physical deconditioning, which describes broader loss of physiological capacity. Spinal muscle health can include features from each of these domains but remains region-specific and function-oriented. Table 1 operationalizes the framework by linking each domain to representative measures, major limitations, and current clinical applicability.

### 4.1. Muscle Quantity and Morphology

MRI, CT, and ultrasonography have described reductions in cross-sectional area (CSA), functional CSA, muscle volume, asymmetry, and segment-specific atrophy in subsets of patients with CLBP. Systematic reviews support an association between low back pain and altered paraspinal morphology, particularly involving the lumbar multifidus, while emphasizing substantial methodological heterogeneity [11,12]. Morphology is therefore informative but incomplete: preserved muscle bulk does not guarantee favorable tissue composition or effective recruitment, and smaller CSA may reflect constitutional factors, aging, body size, activity level, or disease-related change.

### 4.2. Muscle Quality, Fatty Infiltration, and Myosteatosis

Muscle quality refers to tissue characteristics that are not captured by size alone. Fatty infiltration of the lumbar multifidus and other paraspinal muscles has been associated with CLBP, disability, and age-related degeneration, although the magnitude and consistency of these associations vary [13]. MRI-based fat fraction or proton density fat fraction (PDFF), CT attenuation, and qualitative grading can all characterize aspects of composition, but they are not interchangeable measures.

From a broader skeletal muscle perspective, imaging-detected fatty infiltration may represent a regional manifestation of myosteatosis, a condition associated with impaired muscle quality and reduced functional reserve [14,15]. Caution is required when transferring systemic myosteatosis concepts directly to paraspinal muscles because local denervation, disuse, segmental pathology, surgical history, and muscle-specific loading may produce different biological patterns.

### 4.3. Neuromuscular Competence

Paraspinal dysfunction also involves altered activation timing, recruitment strategies, motor control, and endurance. Morphology and neuromuscular function are distinct constructs: a muscle of preserved size may be recruited inefficiently, while an atrophic muscle may retain task-specific activation capacity. Reviews of back muscle pathophysiology emphasize this multidimensionality [6,16]. Electromyography and standardized motor control tasks can provide functional information, but results are task-dependent and influenced by electrode placement, contraction intensity, pain behavior, and normalization procedures.

### 4.4. Muscle-Specific and Level-Specific Considerations

The paraspinal musculature should not be treated as a homogeneous unit. Multifidus has short segmental attachments and a prominent role in intersegmental control; erector spinae contributes more broadly to extension and trunk control; psoas participates in hip flexion and lumbopelvic mechanics and is not a pure spinal extensor; quadratus lumborum contributes to lateral trunk control and load transfer. Their innervation, architecture, loading, and responses to pathology differ. Consequently, a finding in one muscle cannot automatically be generalized to the others.

Recent meta-analytic evidence suggests that abnormalities in CLBP are most consistently demonstrated in the multifidus, particularly at middle-to-lower lumbar levels, whereas findings for erector spinae and psoas are less consistent [13]. Large-scale automated MRI analysis in 9564 UK Biobank participants further demonstrated that paraspinal CSA and intramuscular fat vary with age, sex, BMI, physical activity, muscle, and vertebral level, while differences associated with back pain status were comparatively modest [17]. These observations argue for muscle-specific, level-specific, and covariate-aware interpretation rather than a single global “paraspinal degeneration” metric.

### 4.5. Measurement Approaches and Reproducibility

MRI provides excellent soft-tissue contrast and can quantify CSA, volume, functional CSA, and fat fraction; Dixon or chemical-shift techniques can provide more quantitative compositional measures than visual grading. CT allows opportunistic assessment of CSA and attenuation in Hounsfield units but is affected by acquisition parameters, contrast administration, reconstruction, and radiation-related considerations. Ultrasound offers a low-cost dynamic option for thickness, contraction, and echogenicity, but is operator-dependent and sensitive to probe position, posture, and loading. EMG and functional testing add information on activation and performance but do not directly measure tissue composition [18,19].

Measurement reliability is generally favorable under standardized protocols. A 2026 systematic review and meta-analysis including 73 MRI and 13 CT studies reported excellent reliability for most MRI measures and good-to-excellent reliability for CT measures, while also identifying limited CT evidence and frequent underreporting of reliability methodology [18]. High reliability, however, should not be conflated with cross-platform standardization, diagnostic validity, or established clinical thresholds. Level selection, segmentation boundaries, posture, scanner parameters, normalization to body size, and manual versus automated methods remain important sources of between-study variability.

Automated segmentation may improve scalability and reduce analyst burden, particularly in large imaging datasets [17]. Nevertheless, automated outputs remain dependent on training data, image quality, sequence characteristics, and external validation. At present, no single imaging metric or threshold can be recommended as a stand-alone diagnostic test for CLBP.

## 5. Spinal Muscle Health and Sarcopenia

Sarcopenia and impaired spinal muscle health share biological determinants but should not be considered synonymous. According to the revised European consensus (EWGSOP2), low muscle strength is the primary parameter of sarcopenia; low muscle quantity or quality confirms the diagnosis, and poor physical performance indicates severe sarcopenia [20]. Sarcopenia is a generalized skeletal muscle disorder influenced by aging, inactivity, chronic disease, metabolic dysfunction, inflammation, and nutritional status [21,22].

Spinal muscle health is region-specific and focused on muscles involved in spinal control, load sharing, and movement. A patient may have clinically relevant paraspinal fatty infiltration or neuromuscular impairment without meeting systemic criteria for sarcopenia; conversely, generalized sarcopenia does not imply a uniform degree of paraspinal involvement. Observational data support associations between sarcopenia and low back pain [23], while the emerging concept of spine-specific sarcopenia highlights the need to distinguish regional paraspinal atrophy from generalized muscle loss [24].

The constructs are therefore best viewed as partially overlapping rather than hierarchical. Systemic sarcopenia, frailty, metabolic health, and physical inactivity may influence the biological substrate of paraspinal muscles, whereas regional denervation, pain-related adaptation, segmental degeneration, and local loading may produce spine-specific changes.

## 6. Critical Appraisal of the Evidence

### 6.1. Heterogeneity and Confounding

The literature linking paraspinal muscle characteristics with CLBP is heterogeneous. Definitions of chronicity and case status vary, as do age, sex distribution, BMI, physical activity, occupational exposure, and underlying diagnoses. Studies also differ in the muscle examined, vertebral level, imaging modality, segmentation method, normalization of CSA, and definition of fatty infiltration. These factors can materially alter effect estimates and help explain why apparently similar studies reach different conclusions [11,12,13,17].

The UK Biobank analysis is particularly instructive because age, sex, BMI, and physical activity were independently associated with paraspinal muscle characteristics in a large heterogeneous sample [17]. Observed differences should therefore not be interpreted as disease-specific abnormalities without adequate consideration of these covariates. Similarly, degenerative spinal pathology, radiculopathy, prior surgery, and pain duration may influence muscle characteristics through mechanisms distinct from nonspecific CLBP.

### 6.2. Association Does Not Establish Causation

Most available evidence is cross-sectional. Paraspinal abnormalities may precede pain, develop as a consequence of pain-related disuse or altered motor behavior, or arise from shared determinants such as aging, adiposity, inactivity, denervation, and degenerative spinal disease. These pathways are not mutually exclusive. A 2024 longitudinal MRI study found that most paraspinal CSA and fat-signal-fraction measures remained relatively stable over four months in both LBP and control groups, with only selected muscle- and level-specific differences [25]. The same literature includes longer-term observations in which paraspinal morphology was not clearly associated with subsequent LBP or physical demands [25]. Causal language should therefore be avoided.

### 6.3. Discordance Between Structure, Composition, and Function

Different domains of spinal muscle health do not necessarily change in parallel. A 2025 study combining lumbar MRI with EMG during a standardized lifting task found that muscle size measures were not consistently associated with muscle activity in participants with CLBP, supporting the distinction between morphology, composition, and neuromuscular behavior [26]. This discordance is clinically relevant: a single imaging metric cannot be assumed to represent overall functional capacity.

Negative and weak associations are therefore informative rather than contradictory. They suggest that muscle abnormalities represent one component of a complex CLBP phenotype and that their relevance may depend on muscle, level, task, patient characteristics, and disease context.

## 7. Pain, Deconditioning, and the Biopsychosocial Context

**Figure 1:**
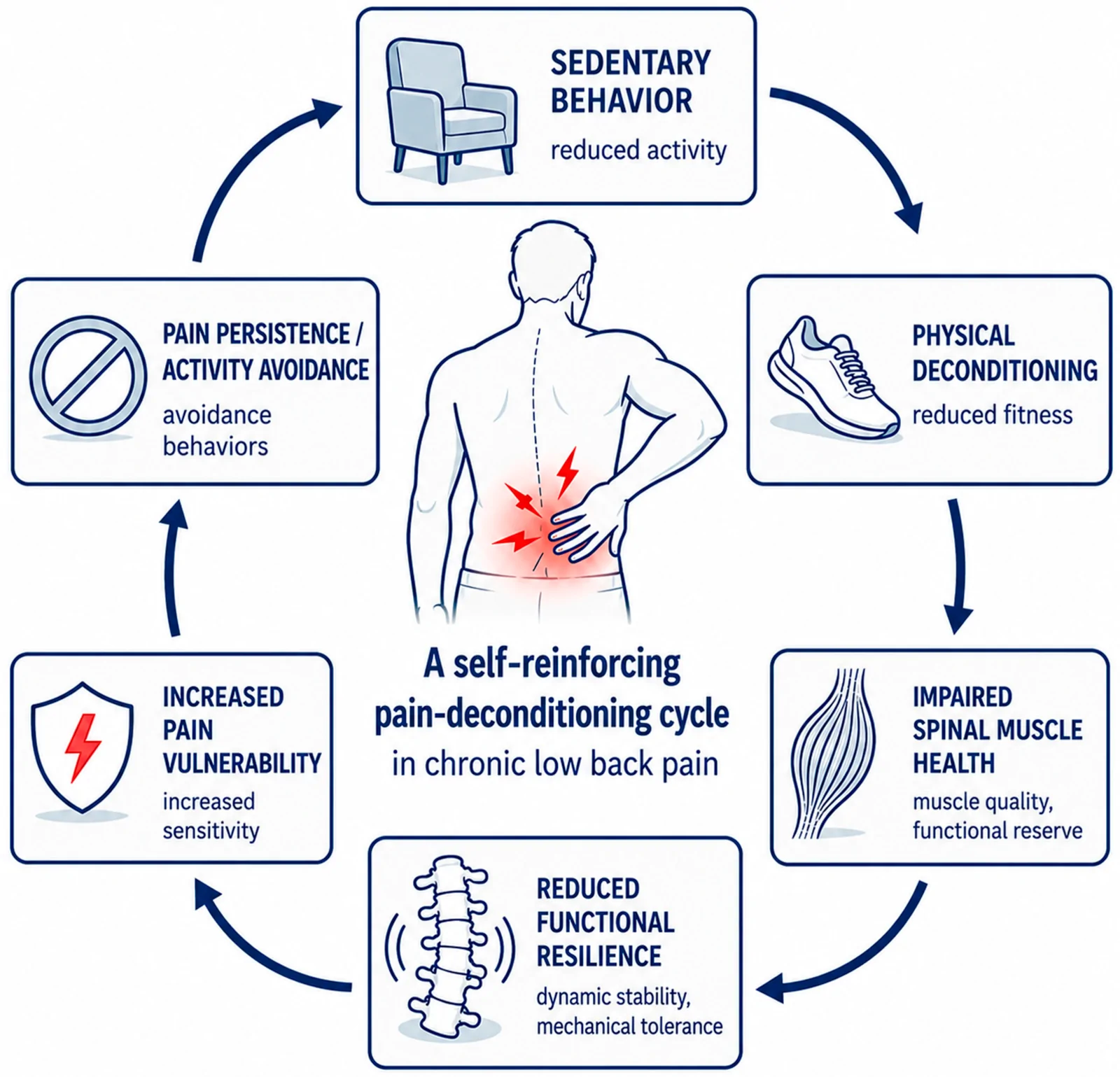
Conceptual representation of the reciprocal pain deconditioning cycle in chronic low back pain. The figure integrates structural pathology, pain, physical activity, spinal muscle health, and functional adaptation. Arrows indicate plausible bidirectional relationships discussed in the literature and should not be interpreted as establishing causality or a universal sequence. Psychosocial and systemic factors may modify multiple pathways within the model.

Pain, reduced activity, muscle dysfunction, and functional decline may interact in a self-reinforcing cycle [27]. Fear of movement and activity avoidance can reduce participation in exercise and daily activities [28], potentially contributing to deconditioning and reduced physiological reserve. Conversely, impaired endurance or neuromuscular control may make movement more demanding and reinforce avoidance. Figure 1 summarizes these reciprocal relationships as a conceptual synthesis rather than a causal model.

Muscle-related factors must nevertheless remain embedded within the broader biopsychosocial model of CLBP [4]. Pain catastrophizing, depression, anxiety, sleep disturbance, occupational demands, social context, and central pain mechanisms may independently influence pain, activity, muscle recruitment, disability, and treatment response. Some may mediate the relationship between pain and physical activity; others may act in parallel. A multidimensional muscle framework is therefore complementary to, not a replacement for, psychosocial assessment.

## 8. Clinical Assessment and Multidimensional Phenotyping

Current evidence does not support spinal muscle health as a validated diagnostic classification or as a stand-alone tool for treatment selection. Rather, available imaging and functional measures provide complementary information on different aspects of paraspinal muscle status [18,19]. Structural imaging remains central when clinically indicated, while existing MRI or CT examinations may also permit assessment of muscle morphology and composition. However, no universally accepted thresholds currently define an abnormal spinal muscle phenotype or indicate a specific treatment [19].

Assessment methods reported in the literature reflect the multidimensional nature of paraspinal muscle evaluation. Muscle quantity can be characterized using cross-sectional area (CSA), functional CSA, or volume, whereas muscle quality can be assessed using MRI-based fat fraction or proton density fat fraction (PDFF), CT attenuation, or standardized qualitative grading [18,19]. Neuromuscular function has been investigated using EMG and task-based motor-control assessment, while endurance, strength, and broader physical conditioning can be evaluated using functional performance measures. These domains provide related but non-interchangeable information and remain influenced by methodological factors including muscle and vertebral level, acquisition protocol, segmentation approach, and patient characteristics [18,19].

Accordingly, current evidence favors interpretation of muscle-related findings as complementary dimensions rather than as a single composite phenotype or validated clinical score. Discordance between morphology, composition, and function should not necessarily be considered contradictory, and the clinical relevance of individual abnormalities remains dependent on the broader structural, functional, and patient context.

## 9. Rehabilitation and Clinical Implications

Exercise therapy improves pain and function in CLBP, although no single exercise mode is consistently superior across all patients [29,30,31]. Current evidence supports viewing rehabilitation as more than an attempt to increase muscle size. Depending on the clinical presentation, relevant targets may include strength, endurance, motor control, movement confidence, aerobic conditioning, physical activity, and restoration of meaningful function.

Importantly, clinical improvement should not be assumed to require normalization of muscle morphology or fatty infiltration. A systematic review of exercise interventions found no consistent evidence that paraspinal fatty infiltration is reversible with exercise and concluded that larger, methodologically consistent trials are needed [32]. More recently, a 2025 randomized trial comparing 10 weeks of aquatic exercise with standard care in 34 participants found no significant between-group time-by-treatment interaction for multifidus or erector spinae volume or fatty infiltration, although strength improved and selected within-group morphological changes and correlations with patient-reported outcomes were observed [33]. These findings reinforce the distinction between imaging biomarkers and clinical benefit.

Accordingly, the clinical interpretation of muscle-related findings should remain conservative. Existing MRI or CT may provide contextual information on muscle quantity or composition without requiring additional imaging solely for this purpose. Simple strength, endurance, and functional tests may identify modifiable limitations. Ultrasound can provide accessible dynamic assessment where local expertise and standardized protocols are available. However, no evidence currently supports prescribing a specific exercise program solely on the basis of a paraspinal imaging phenotype, and no validated cut-offs exist for routine treatment selection.

## 10. Future Research Priorities

Several steps are required before spinal muscle health can become clinically actionable. First, assessment protocols require greater standardization across imaging acquisition, vertebral level, segmentation boundaries, normalization procedures, and reporting. Reliability can be high within a protocol [18], but reproducibility across centers and clinically meaningful thresholds remain insufficiently established.

Second, longitudinal studies should determine temporal relationships among pain, activity, muscle composition, neuromuscular function, and systemic health. Studies should stratify or adjust for age, sex, BMI, physical activity, spinal pathology, and psychosocial factors and should avoid treating different paraspinal muscles as equivalent. Third, interventional trials should test whether changes in specific muscle domains mediate or predict changes in pain, disability, quality of life, and return to activity, rather than assuming that imaging normalization is a necessary therapeutic target.

Fourth, automated segmentation and quantitative imaging may enable large-scale phenotyping and improve measurement efficiency [17,34]. These tools require external validation across scanners, sequences, institutions, and patient populations. Finally, prospective studies should test whether integrating muscle-related information with structural, neurological, systemic, functional, and psychosocial variables provides incremental prognostic value and, crucially, whether phenotype-guided interventions improve outcomes compared with standard clinical assessment.

## 11. Conclusions

CLBP is a multifactorial condition whose clinical presentation cannot be explained by structural pathology alone. Paraspinal muscle abnormalities encompass distinct dimensions of quantity, tissue composition, neuromuscular competence, endurance, and conditioning. The available evidence supports clinically relevant associations, particularly for selected multifidus measures, but findings are heterogeneous across muscles, spinal levels, populations, and measurement methods.

Spinal muscle health can be considered as a multidimensional, region-specific construct encompassing complementary aspects of muscle quantity, tissue composition, neuromuscular competence, endurance, and conditioning. Such an approach may help avoid reducing muscle status to a single measure and place regional muscle findings within their systemic, functional, and biopsychosocial contexts. Current evidence does not establish a universal causal pathway from muscle abnormality to CLBP, nor does it support imaging-based treatment selection or universal cut-offs.

Future progress will depend on standardized assessment, longitudinal and interventional research, and demonstration of incremental clinical value. Until then, muscle-related measures should be interpreted as complementary to established structural and clinical assessment and within the broader patient-specific functional and biopsychosocial context.

## Figures and Tables

**Table 1:** Operational domains of spinal muscle health, representative assessment approaches, principal limitations, and current clinical applicability.

Domain	Representative Measures	What It May Capture	Major Limitations	Current Clinical Applicability
Muscle quantity	CSA; functional CSA; volume; asymmetry	Muscle size, regional atrophy, side-to-side differences	Strongly influenced by sex, body size, vertebral level, normalization, and segmentation; size does not equal function	Research and contextual interpretation; no validated diagnostic cut-offs
Muscle quality	MRI fat fraction/PDFF; CT attenuation; standardized qualitative fatty infiltration grading	Tissue composition and intramuscular fat/myosteatosis-related features	Sequence/scanner and CT protocol dependence; grading methods are not interchangeable; thresholds not standardized	Promising quantitative biomarker domain; mainly research/contextual use
Neuromuscular competence	Surface/fine-wire EMG; activation timing; task-based motor control testing	Recruitment, timing, coordination, task-specific activation	Task-, electrode-, pain-, and normalization-dependent; limited routine standardization	Primarily research/specialist assessment
Endurance and strength	Trunk extensor endurance; dynamometry; standardized functional tests	Performance capacity and fatigability	Not muscle-specific; influenced by pain, motivation, fear, and general conditioning	Feasible adjunct to routine functional assessment
Conditioning/functional reserve	Physical performance tests; activity monitoring; participation measures	Whole-person adaptive capacity and real-world function	Influenced by cardiovascular, systemic, behavioral, and psychosocial factors	Clinically feasible but not specific to paraspinal muscle health

## Data Availability

No new data were created or analyzed in this study. Data sharing is not applicable to this article.
